# Complete Genome Sequence of Strain BW-2, a Magnetotactic Gammaproteobacterium in the Family *Ectothiorhodospiraceae*, Isolated from a Brackish Spring in Death Valley, California

**DOI:** 10.1128/MRA.01144-19

**Published:** 2020-01-02

**Authors:** Corey Geurink, Christopher T. Lefevre, Caroline L. Monteil, Viviana Morillo-Lopez, Fernanda Abreu, Dennis A. Bazylinski, Denis Trubitsyn

**Affiliations:** a School of Medicine, University of Nevada at Las Vegas, Las Vegas, Nevada, USA; b Aix-Marseille University, CNRS, CEA, UMR7265, Institute of Biosciences and Biotechnologies of Aix Marseille, CEA Cadarache, Saint-Paul-lès-Durance, France; c School of Life Sciences, University of Nevada at Las Vegas, Las Vegas, Nevada, USA; d Universidade Federal do Rio de Janeiro, Rio de Janeiro, Brazil; e Department of Biological and Environmental Sciences, Longwood University, Farmville, Virginia, USA; University of Southern California

## Abstract

We report the complete 4.1-Mb genome sequence of strain BW-2, a magnetotactic, sulfur-oxidizing rod, belonging to the family *Ectothiorhodospiraceae* of the class *Gammaproteobacteria*, that biomineralizes membrane-bounded magnetite nanocrystals in its magnetosomes. This genome sequence, in comparison with those of other magnetotactic bacteria, is essential for understanding the origin and evolution of magnetotaxis and magnetosome biomineralization.

## ANNOUNCEMENT

Magnetotactic bacteria (MTB) synthesize intracellular membrane-bound magnetic nanocrystals termed magnetosomes (1). Chains of magnetosomes cause cells to align along the earth’s geomagnetic field lines and are thought to function in aiding MTB in locating and maintaining an optimal position for survival and growth (i.e., the oxic-anoxic interface) in aquatic environments (2). Strain BW-2 is a magnetotactic, sulfur-oxidizing rod which is motile via a polar bundle of flagella that biomineralize cuboctahedral magnetite nanocrystals (3). Strain BW-2 was isolated from mud and water samples collected from a brackish spring at Badwater Basin in Death Valley National Park in California (3). Here, we present the complete genome sequence of strain BW-2, the only completed genome sequence of a magnetotactic gammaproteobacterium. This sequence, in comparison with those of other MTB, is essential for elucidating the origin and evolution of magnetotaxis and magnetosome biomineralization (4).

Two axenic cultures of strain BW-2, inoculated from the same source, were grown in 2-liter flasks in a semisolid medium with an O_2_ concentration gradient, with the addition of sulfide (5, 6), and were combined after harvesting by centrifugation. Genomic DNA was purified and DNA libraries were prepared using the following commercial kits, according to the manufacturers’ standard protocols: DNeasy blood and tissue kit (Qiagen), QIAquick PCR purification kit (Qiagen), Zymo Clean & Concentrator kit (Zymo Research), Nextera XT kit (Illumina), and Kapa library preparation kit (Kapa Biosciences).

Genome sequencing was performed with 11.8 million reads (800× coverage) of the paired-end sequence with 300-bp inserts (Illumina MiSeq) and 242,620 long reads (333× coverage; *N*_50_, 8.9 kb) with three PacBio RS single-molecule real-time cells (Pacific Biosciences) (7). Default parameters were used for all sequencing analysis software. Illumina-generated raw data were processed using the CLC Bio Genomics Workbench 8.5.1 (Qiagen) prepare raw data workflow, with a quality limit of 0.05, an ambiguous limit of 2, and automatic read-through adapter trimming. Assembly of Illumina-generated reads resulted in the following numbers of contigs: 479 contigs with SPAdes 3.5.0 (8), 992 contigs with Velvet 1.2 (9), and 168 contigs with CLC Bio Genomics Workbench 8.5.1. PacBio reads were used to complete scaffolding, using CLC Bio Genomics Workbench 8.5.1 with Genome Finishing Module 1.5.2. Automated annotation with MicroScope 3.1 (10) was used to confirm the presence of genes of interest. The standard PGAP annotation (11) is available at GenBank.

The genome of BW-2 consists of a single circular chromosome, 4,103,727 bp long, with a G+C content of 52.6%; 3,791 predicted coding DNA sequences, 41 tRNAs, and 2 identical sets of 5S/16S/23S rRNAs were identified. Phylogenetically, strain BW-2 belongs to the order *Thiotrichales* in the family *Ectothiorhodospiraceae* (3). Based on the highest average amino acid identity (AAI) value of 54.01%, which was calculated pairwise using the ANI/AAI-Matrix online service (http://enve-omics.ce.gatech.edu/g-matrix) and default parameters (12), and data from a 16S rRNA gene comparison of <90% sequence identity with other related strains for which genomes are not yet available (e.g., Thiohalospira alkaliphila, Thiogranum longum, and *Granulosicoccus* sp.), strain BW-2 appears to represent a novel genus.

Based on annotation using MicroScope, the BW-2 genome contains genes necessary for magnetosome biomineralization, autotrophy via the Calvin-Benson-Bassham cycle, oxidation of reduced sulfur compounds, including the *SOX* and *Dsr* systems, and nitrogen fixation. This genome sequence has proved essential for understanding the evolution of magnetotaxis and the metabolic potential of magnetotactic gammaproteobacteria.

### Data availability.

The annotated genome sequence for strain BW-2 has been deposited in GenBank under accession number CP032507. The PacBio and Illumina raw sequencing reads are available in the Sequence Read Archive database under accession numbers SRX6815148 and SRX5884006, respectively.
